# Invincible DNA tethers: covalent DNA anchoring for enhanced temporal and force stability in magnetic tweezers experiments

**DOI:** 10.1093/nar/gku677

**Published:** 2014-08-12

**Authors:** Richard Janissen, Bojk A. Berghuis, David Dulin, Max Wink, Theo van Laar, Nynke H. Dekker

**Affiliations:** Department of Bionanoscience, Kavli Institute of Nanoscience, Faculty of Applied Sciences, Delft University of Technology, Lorentzweg 1, 2628 CJ Delft, The Netherlands

## Abstract

Magnetic tweezers are a powerful single-molecule technique that allows real-time quantitative investigation of biomolecular processes under applied force. High pulling forces exceeding tens of picoNewtons may be required, e.g. to probe the force range of proteins that actively transcribe or package the genome. Frequently, however, the application of such forces decreases the sample lifetime, hindering data acquisition. To provide experimentally viable sample lifetimes in the face of high pulling forces, we have designed a novel anchoring strategy for DNA in magnetic tweezers. Our approach, which exploits covalent functionalization based on heterobifunctional poly(ethylene glycol) crosslinkers, allows us to strongly tether DNA while simultaneously suppressing undesirable non-specific adhesion. A complete force and lifetime characterization of these covalently anchored DNA-tethers demonstrates that, compared to more commonly employed anchoring strategies, they withstand 3-fold higher pulling forces (up to 150 pN) and exhibit up to 200-fold higher lifetimes (exceeding 24 h at a constant force of 150 pN). This advance makes it possible to apply the full range of biologically relevant force scales to biomolecular processes, and its straightforward implementation should extend its reach to a multitude of applications in the field of single-molecule force spectroscopy.

## INTRODUCTION

Single-molecule techniques have become increasingly important in the last two decades, as they have permitted detailed insights to biological processes that are not readily apparent in ensemble measurements. They also provide access to novel parameters such as force, which plays a fundamental role in a variety of biological processes ranging from cellular motility to the replication, repair and segregation of DNA (1,2). Examples of single-molecule methods capable of applying force to biological systems include atomic force spectroscopy (AFM), optical tweezers and magnetic tweezers (2,3). The magnetic and optical tweezers methods have garnered particular attention as they provide high temporal, spatial and force sensitivity in a range from hundreds of femtoNewtons to tens of picoNewtons (pN), and, in contrast to AFM, they are also capable of measuring and applying torque (4–7). More recent developments in magnetic tweezers also include the possibility of measuring multiple samples simultaneously (multiplexing) (8–10), allowing for a broad, statistically sound characterization of biomolecular machines at the single-molecule level.

Studies of biomolecular processes such as DNA unzipping (11), polymerase dynamics (12), nucleosome unwrapping (13), protein unfolding (14), conformational changes such as the overstretching transition of nucleic acid structures (15) and viral genome packaging motors (16) have particularly stringent requirements on the applied forces, which need to exceed several tens of pN. For example, the dsDNA B-S conformational transition is only observed at pulling forces of ∼65 pN (17). However, as the lifetime of the non-covalent bonding typically employed for the anchoring of biomolecules in magnetic or optical tweezers decreases with applied force (18–20), such studies are limited to short measurement times in the order of minutes. As a consequence, the low amount of data that can be collected from individual tethers can hamper reliable characterization of the biomolecular process of interest. To allow measurements over longer timeframes and broader force spectra, the anchoring of biomolecules in tweezers experiments should employ strong chemical bonding.

Despite significant developments in tweezers instrumentation and methodology in recent years (4–7,21), the chemistry of the biomolecular anchoring techniques has remained largely unaltered (22–24). Focusing more specifically on magnetic tweezers, typically DNA constructs containing digoxigenin (DIG)-modified nucleotides (incorporated by enzymatic polymerase chain reaction (PCR)) are bound onto surfaces to multiple non-specifically adsorbed anti-digoxigenin IgG antibodies (anti-DIG) (7). The attachment of superparamagnetic beads to DNA tethers is mainly realized by biotin:streptavidin linkages (7,9,21). While this methodology permits a rapid and reliable DNA attachment, allowing it to become widespread, known limitations exist. For example, a single DIG:anti-DIG interaction has a higher thermodynamic dissociation constant (*K*_d_ = 1.2 × 10^−9^ M) and significantly lower force stability (*F*_max_ ∼25 pN) (25) compared to a biotin:streptavidin complex, for which *K*_d_ = 10^−14^ M and *F*_max_ ∼200 pN (26). While multiple of these non-covalent ligand:receptor bondings may be employed to extend the force stability and lifetime of tethered DNA (7,9,21), the facilitated dissociation of bonds due to the application of external forces (27) continues to constrain both parameters.

**Figure 1.**
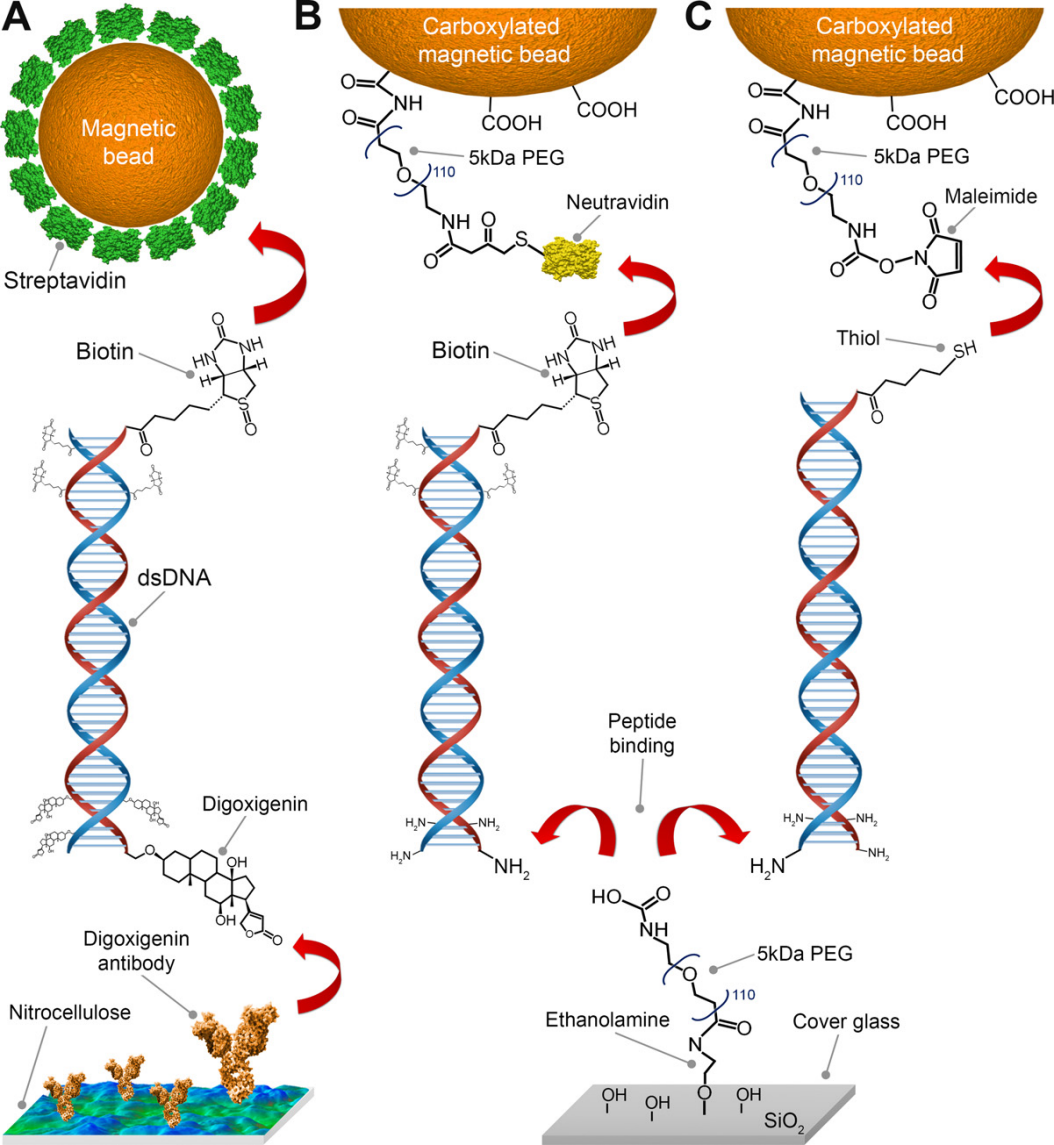
Schematic overview of DNA anchoring strategies used in this study. (**A**) Tethering of DNA in magnetic tweezers by coupling a multiply biotinylated handle on one extremity of the DNA to a streptavidin-coated magnetic bead, and by binding a handle containing multiple digoxigenin molecules on the other extremity of the DNA to anti-digoxigenin IgG antibodies adsorbed on nitrocellulose. (**B** and **C**) Tethering of one DNA extremity via covalent coupling NH_2_-enriched handles on the DNA to a surface. The other DNA extremity can then be bound to either (B) neutravidin-coated magnetic beads using labeled DNA handles containing multiple biotins or to (C) maleimide-labeled magnetic beads using a single DNA 5′ thiol-modification. In both cases (B and C) the magnetic bead coatings include PEG polymers that serve both as a passivation layer and as a covalent crosslinker.

In this work, we achieve the covalent anchoring of DNA tethers for use in magnetic tweezers (Figures 1 and 3A). To do so, we introduce the application of ethanolamine and heterobifunctional poly(ethylene glycol) (PEG) linkers that allow the covalent binding of DNA molecules to either glass or magnetic beads (28,29). This development relies on the ability to selectively change the functional moieties of PEG-crosslinkers (28). We quantitatively characterize both the PEG-based surface passivation and DNA-tether stability in the magnetic tweezers. Our studies of the attachment stabilities of DNA tethers, probed by dynamic force spectroscopy and lifetimes experiments, demonstrate that DNA molecules anchored via covalent coupling to PEG-coated surfaces and magnetic beads withstand pulling forces up to 150 pN and exhibit lifetimes exceeding 24 h under constant force loads of 45 and 150 pN. Our use of amphiphilic PEG polymers, widely used in single-molecule AFM force experiments (29–32), provides the additional benefit of excellent biocompatibility (33–36) and, as we experimentally demonstrate, increased suppression of non-specific adhesions (37,38) over a range of ionic conditions compared to the commonly applied DNA-tethering method using nitrocellulose surfaces and streptavidin-coated beads. These advances overcome the current limitations on the applicable force and lifetime stabilities of DNA tethers, thereby vastly extending the impact of future magnetic and optical tweezers studies.

**Figure 2.**
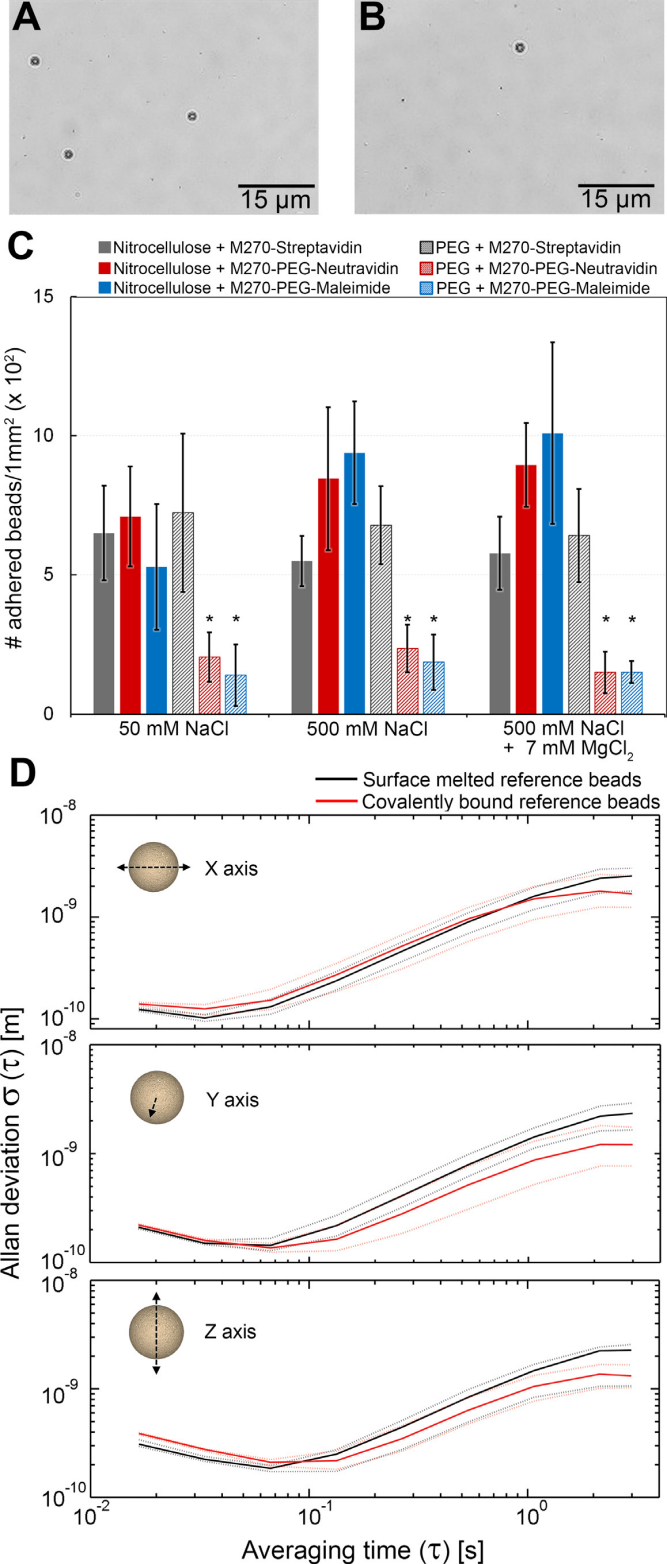
Non-specific adhesion and spatial stability of beads. (**A** and **B**) Images of a flow cell with non-specifically adhered magnetic beads. (**C**) The number of adhered magnetic beads is summarized in the histogram for different combinations of bead-surface for a variety of buffer conditions. The error bars represent the mean standard deviations and the asterisks denote the significance threshold level of the applied analysis of variance (1-way ANOVA; *P < 0.05) for each buffer condition. (**D**) The position stability of reference beads affixed via surface-melting (black) or covalent attachment (red) is analyzed by computing their respective Allan deviations (48) as a function of time in all three dimensions. For each attachment method, the median of *n* = 25 reference beads is presented as continuous lines and the 25th and 75th percentiles as dashed lines.

**Figure 3.**
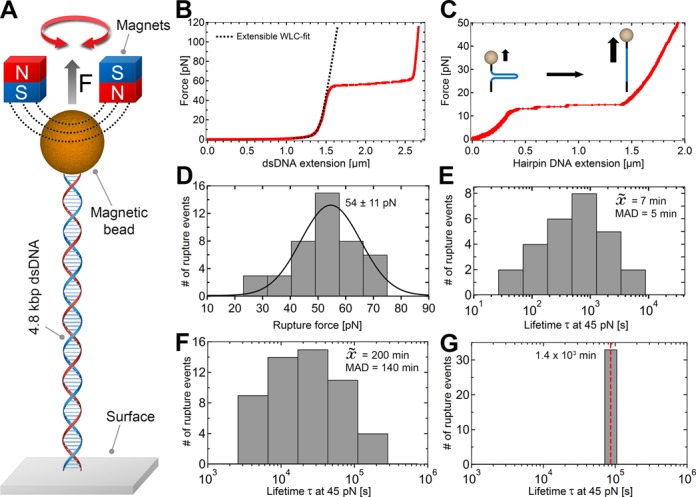
DNA stability under high forces. (**A**) DNA tethers were stretched by applying a magnetic field via a pair of superparamagnetic magnets. (**B**) Force-extension plot for a covalently anchored linear 4.8 kbp dsDNA tether. The black dashed line is a fit of the extensible worm-like-chain fit to the data. At ∼60 pN, the overstretching transition of DNA is observed. (**C**) Force-extension plot for a covalently surface-bound single 1 kbp hairpin DNA construct. (**D**) Histogram of measured rupture forces (*n* = 43) via DFS with constant loading rate of 10pN/s up to 150 pN and applied Gaussian fit (black curve). (**E**) Histogram of measured lifetimes at constant pulling force of 45 pN (*n* = 37) for dsDNA tethers anchored via DIG:anti-DIG to the surface and streptavidin:biotin to the magnetic bead. (**F** and **G**) Histograms of observed lifetimes at applied forces of 45 pN for DNA molecules anchored covalently to the surface alone (*n* = 52) (F) and anchored covalently to both surface and bead (*n* = 32) (G). The median of tether lifetimes in (E and F) is denoted within the histogram panels as $\tilde X$ with the corresponding median absolute deviation (MAD). The red dashed line in (G) is meant to indicate that the measured lifetimes are lower bounds, as the experiment itself was terminated after 24 h.

## MATERIALS AND METHODS

### Materials

#### Beads

Streptavidin-coated and carboxylated superparamagnetic beads (M-270 DynaBeads, LifeTechnologies, USA) with a diameter of 2.8 μm were used within this study. Commercially available amine-coated polystyrene beads (Polysciences GmbH, Germany) with a diameter of 3μm were used as reference beads fixed onto the functionalized glass surfaces.

#### DNA constructs

Linear dsDNA and single DNA hairpin constructs, each with an enzymatically ligated dsDNA handle at both ends, were synthesized as samples.

For the chemical anchoring stability characterization and tether lifetime survey, linear double-stranded DNA constructs with a total length of 4.8 kbp were synthesized based on a 3.6 kbp fragment of the pRL-SV40 plasmid (Promega, USA), digested with BamHI and XbaI and ligated in a second step to the 600 bp dsDNA handles, as previously described (7,39). To create handles, a 1.2 kb fragment from pBluescript (Stratagene, USA) was amplified by PCR in the presence of Biotin-16-dUTP (Roche Diagnostics, Switzerland), Digoxigenin-11-dUTP (Roche Diagnostics, Switzerland) or Aminoallyl-dUTP (Thermo Scientific, USA) using forward primer 5′-GACCGAGATAGGGTTGAGTG and reversed primer 5′-CAGGGTCGGAACAGGAGAGC. Prior to enzymatic ligation via T4 DNA ligase (New England Biolabs Ltd., UK), the handles were digested with either BamHI or XbaI. Thiol-labeled DNA handles were produced by PCR using the same template and primers with the same sequence as mentioned above, but containing a 5′ thiol-labeled nucleotide (Biolegio B.V, The Netherlands). Three different full-length construct combinations used in this study were assembled as shown (Figure 1).

To prepare a DNA hairpin construct, a 1 kb Lambda phage dsDNA fragment (LifeTechnologies, USA) was amplified by PCR using the forward primer 5′-CTGCGGTCTCGTTGCTTACCGTCACCAGAAATTACCGTCAC and reversed primer 5′-CCATCTTGGTCTCCTAGGTTTTTAGCAGCGAAGCGTTTGATAAG, digested with BsaI and ligated at one DNA terminus with a 42 bp oligonucleotide to form a U-turn (Table 1). Biotin-16-dUTP and Aminoallyl-11-dUTP enriched handles (see above) were ligated to double-stranded spacer oligonucleotides (Biolegio B.V., The Netherlands; Table 1). These handle/spacer constructs were allowed to anneal in order to form a short (50 bp) double-stranded stem with a *BsaI* overhang, which were ligated to the free accessible terminals of the 1 kb dsDNA-hairpin fragment.

**Table 1.** Sequences of the oligonucleotides used for the synthesis of the 1 kbp hairpin constructs

Oligonucleotides	Sequences
U-turn	5′CCTAAGCTCGCCGAGGCGAGCGAAAGCTCGCCTCGGCGAGCT
Upper handle, forward	5′GGCCAACCAAGTCATTCTGAGAATAGTGTATGCGGCGACCGAGTTGCTCTTGCCATGCTCTTTACAACCGGTTGACTGCTTCAGGGGTCGATCCCGCTTTGTAC
Upper handle, reversed	5′GGCAAGAGCAACTCGGTCGCCGCATACACTATTCTCAGAATGACTTGGTT
Lower handle, forward	5′GCAAGTACAAAGCGGGATCGACCCCTGAAGCAGTCAACCGGTTGTAAAGAGCATCGATCGTTGTCAGAAGTAAGTTGGCCGCAGTGTTATCACTCATGGTTATGCCAGATGGTAAGCCCTCCCGTATCGTAGTTATCTACACGACGGGGAGTCAGGCAACTATGGATGAACGA
Lower handle, reversed	5′GATCTCGTTCATCCATAGTTGCCTGACTCCCCGTCGTGTAGATAACTACGATACGGGAGGGCTTACCATCTGGC

#### Poly(ethylene glycol) crosslinker

For surface passivation and covalent DNA coupling procedures, a mixture of mono- and heterobifunctional PEG linker of 5 kDa (∼110 monomers) were used. For covalent amino-labeled DNA anchoring, NHS-PEG-COOH and NHS-mPEG (LaysanBio, USA) were selected as surface linker; for PEGylation of the superparamagnetic beads NH_2_-PEG-maleimide (Nanocs, USA), NH_2_-PEG-SH and NH_2_-mPEG (IRIS Biotech, Germany) were used.

### Methods

#### Functionalization of superparamagnetic beads

For the functionalization of magnetic beads, we started with commercially available carboxylated superparamagnetic beads. For a final volume of 1 ml with a bead concentration of 3 mg/ml (∼ 2 × 10^8^ beads), 100 μl of the stock solution was used for any functionalization strategy (Figure 1).

The vials with the stock solution were shaken for 30 min using a tilting mixer to avoid colloidal aggregates prior to the functionalization steps. Afterward, 100 μl of the carboxylated bead stock solution was transferred to a 1 ml Eppendorf tube and placed in a magnetic holder (DynaMag-5, LifeTechnologies, USA) for 2 min, after which the supernatant was discarded. The beads were washed twice with 1 ml 100 mM MES (2-(*N*-morpholino)ethane sulfonic acid, Sigma, USA) buffer (pH 4.7) for 10 min with a tube shaker at 1200 rpm (Thermomixer, Eppendorf, USA), and the supernatant was discarded after each washing step which includes a 2 min incubation in the magnetic holder. The beads were resuspended in 100 μl MES buffer and mixed with a 100 μl MES solution containing 4 mg of NH_2_-PEG-SH (for binding neutravidin in a second step) or NH_2_-PEG-maleimide (depending on the DNA anchoring strategy, see Figure 1), 8 mg NH_2_-mPEG and 20 mg EDC (1-Ethyl-3-(3-dimethylaminopropyl)carbodiimide, Sigma, USA). The components were mixed thoroughly for 2 min with a vortex, and afterward the bead solution was sonicated for 2 h in an ultrasonic bath (Bransonic B1510, Branson, USA) with an additional 30 s mixing step every 20 min. Following the PEGylation reaction, the functionalized beads were washed three times with 1 ml PBS (phosphate buffered saline, Sigma, USA) buffer (pH 7.4) containing 2% BSA (bovine serum albumin, Sigma, USA). Each washing step consists of 10 min of shaking at 1200 rpm followed by placement of the tube in the magnetic holder for 2 min, after which the supernatant was discarded. The functionalized magnetic beads were resuspended in 1 ml PBS buffer (2% BSA, pH 7.4).

For the coating of magnetic beads with neutravidin, PEGylated beads with NH_2_-PEG-SH were resuspended in 100 μl sodium phosphate (SP, pH 7) buffer (Sigma, USA) and mixed with 100 μl SP buffer containing 100 μg of maleimide-labeled neutravidin (Pierce, USA). After vortexing the mixture for 1 min, the coupling reaction was carried out in the tube shaker at 1200 rpm for 2 h. Afterward, the neutravidin-coated beads were washed three times with 1 ml PBS buffer (2% BSA, pH 7.4) for 10 min at 1200 rpm, constantly discarding the supernatant after placing the tube in the magnetic holder for 2 min and after each mixing instance. The magnetic beads were resuspended in 1 ml PBS buffer (2% BSA, pH 7.4).

All functionalized magnetic beads were stored at 4°C and used within 30 days following bead functionalization. No noticeable decreases in the DNA-coupling efficiency were observed during this period.

#### Functionalization of flow cell surfaces

Borosilicate cover glasses (#1, Menzel GmbH, Germany) with dimensions of 24 × 60 mm serve as the flow cell surface for DNA anchoring. The glasses were placed into a Teflon holder and incubated in a 5% (V/V) aqueous Hellmanex III (Hellma GmbH, Germany) solution and sonicated for 20 min at 40°C. After washing thoroughly with water, the glasses were covered in water and sonicated for further 20 min. After another washing step with water, the glass surfaces were dried in a nitrogen flow (28,29). The pre-activation of the glass surfaces to generate a high density of silanole groups—which allows a homogeneous surface functionalization—was realized by applying oxygen plasma (Plasma-PREEN I, Plasmatic Systems Inc., USA) for 2 min with an oxygen flow rate of 2.5 SCFH and a power of 200 W.

For the coating of glass surfaces with nitrocellulose, nitrocellulose membrane paper (Invitrogen, USA) was dissolved (1% m/V) in acetic acid pentyl ester (Sigma, USA) by mixing the components for 1 h at 250 rpm in a tube shaker at 35°C. Prior to polymer coating, polystyrene beads with a diameter of 3 μm were melted to the previously cleaned cover glasses to serve as reference beads for the magnetic tweezers experiments. For that purpose, 3 μl of a 250× dilution of the polystyrene bead stock solution in ethanol (Sigma, USA) was added to the cover glasses and spread evenly on the surface with the lateral side of the pipette tip. For bead fixation, the cover glasses were heated to 150°C for 3 min on a ceramic heat plate (PC-420D, Corning, USA). Afterward, the prepared nitrocellulose solution was diluted 10×, and 3 μl of it was spread evenly onto each surface and cured by evaporation at 90°C for 1 min.

The glass surfaces for covalent DNA attachment were aminated by esterification of ethanolamine with surface silanole groups (28,29). The clean cover glasses were incubated overnight in a 5 M ethanolamine hydrochloride (EA, Sigma, USA) solution in anhydrous dimethyl sulfoxide (DMSO, Sigma, USA). The dissociation of EA in DMSO was performed at 65°C for ∼1 h, accompanied by some occasional shaking to ensure complete dissolution. After the surface amination, the cover glasses were washed thoroughly with water and dried with a nitrogen stream.

The functionalized glass surfaces were afterward used for flow cell assembly as previously described (7). In brief, a double layer of parafilm spacer was placed onto the functionalized surfaces, and the flow cell was closed by a second coverslip on top containing inlet and outlet apertures for fluidic couplings. The assembly was performed by melting the parts together at 90°C for 30 s.

#### Evaluation of non-specific magnetic bead adhesion

To quantify the non-specific interactions between functionalized magnetic beads and coated surfaces, we counted the number of non-specific adhered beads per field of view (∼260 × 190 μm) of the complementary metal-oxide-semiconductor (CMOS) camera for each different bead-surface configuration. Experimentally, chemically functionalized magnetic beads with a concentration of 6 × 10^7^ beads/ml were incubated on the functionalized surfaces for 30 min in PBS/BSA buffer (1% BSA, pH 7.4), followed by washing with 3 ml of the same buffer until no more mobile beads were observed during flow cell flushing. For each bead-surface configuration, 20 of such areas were investigated to calculate the average number and standard deviation of non-specifically adhered beads. The data was additionally evaluated by a Kruskal–Wallis one-way analysis of variance (ANOVA), followed by a Tukey's post-hoc test (40).

#### DNA anchoring and magnetic bead tethering

The DNA constructs with different functional moieties were anchored to the functionalized surfaces according to different chemical approaches (Figure 1).

As a standard method, DNA constructs with biotin and digoxigenin (DIG) labeled handles were surface-anchored via anti-DIG antibodies and coupled to streptavidin-coated superparamagnetic beads as previously described (7,9,21). In detail, first a volume of 50 μl PBS buffer (pH 7.4) containing 100 μg/ml of anti-DIG antibodies (Roche, USA) was flushed into the flow cell and incubated for 1 h. Afterward, the flow cell was washed with 1 ml PBS/BSA buffer (1% BSA, pH 7.4) and incubated with this solution for another hour to passivate the surface, decreasing the non-specific adhesion of biomolecules and magnetic beads. After another washing step with 500 μl PBS/BSA buffer, 16 pM of the DNA construct in 100 μl PBS/BSA buffer was incubated within the flow cell for 1 h for surface anchoring, followed by a washing step using 1 ml PBS/BSA buffer. A volume of 100 μl PBS/BSA buffer containing 100 μg of streptavidin-coated magnetic beads was then incubated for another hour within the flow cell to tether the DNA. A final washing step with PBS/BSA buffer was applied until all non-tethered beads were removed.

To covalently anchor amino-labeled DNA to the surface via peptide binding (Figure 1B and C), the previously aminated surfaces were PEGylated. To achieve this, a mixture of 3.5 mg NHS-PEG-COOH and 6.5 mg NHS-mPEG in 500 μl MES/EDC buffer (100 mM MES, 50 mM EDC, pH 4.7) was prepared and incubated in the flow cell for 1 h. After washing with 3 ml water, a concentration of 16 pM amino-labeled DNA constructs in 100 μl MES/EDC buffer was mixed with 1.5 × 10^4^ amino-labeled polystyrene reference beads and added to the flow cell for 1 h. After covalent anchoring of DNA and reference beads to the PEG surface, the flow cells were washed with 1 ml water, followed by incubation in PBS/BSA buffer for another hour as an additional passivation step. The flow cells were then washed with 500 μl PBS/BSA buffer and bead tethering was performed in one of two possible ways, depending on the functional moieties included on the free accessible end of the DNA constructs.

Thus, to bind DNA constructs that contain biotinylated handles to magnetic beads, 100 μg of neutravidin-coated magnetic beads in 100 μl PBS/BSA buffer was incubated within the flow cell for 1 h. Alternatively, to covalently tether sulfhydryl-labeled DNA to magnetic beads, the thiol groups were reduced prior by incubation with 2 mM DTT (Dithiothreitol) in PBS buffer (pH 7) for 1 h, followed by a washing step using 1 ml PBS buffer. Afterward, 100 μg of maleimide-modified magnetic beads in 100 μl PBS/BSA buffer was added to the flow cell and incubated for 1 h. In both cases, a final washing step was applied using PBS/BSA buffer until all non-tethered beads were removed.

#### Magnetic Tweezers experimental configuration

The magnetic tweezers implementation used in this study has been described previously (9,21). Briefly, light transmitted through the sample was collected by a 100× oil-immersion objective (Apochromat 100×, NA = 1.25, Olympus, USA) and projected onto a 4.1 MP CMOS camera (Falcon 4M60, Teledyne Dalsa, Canada) with a sampling frequency of 60 Hz. The applied magnetic field was generated by a pair of vertically aligned permanent neodymium-iron-boron magnets (SuperMagnete, Switzerland) separated by a distance of 300 μm, suspended on a motorized stage (M-126.PD2, Physik Instrumente, Germany) above the flow cell. Additionally, the magnet pair could be rotated about the illumination axis by an applied DC servo step motor (C-149.PD, Physik Instrumente, Germany).

Image processing of the collected light allowed us to track the real-time position of both surface-attached reference beads and superparamagnetic beads coupled to DNA tethers in three dimensions. This was achieved using a cross-correlation algorithm realized with custom-written software in LabView (2011, National Instruments Corporation, USA). After subtraction of the relative reference bead position to correct for instrumental drift, the *x*, *y* and *z* position of the DNA-tethered beads were determined with a spatial accuracy of <3 nm. The upward stretching forces on the DNA tethers by the superparamagnetic beads were calibrated from the bead Brownian motions, whereby spectral corrections were employed to correct for camera blur and aliasing (41).

#### Tether rupture force and lifetime measurement

To statistically compare the mechanical stability between DNA molecules tethered using the three different chemical anchoring strategies (Figure 1), we employed two approaches: we applied dynamic force spectroscopy to evaluate the DNA tether rupture force, and we performed force clamp experiments at 45 and 150 pN to evaluate DNA tether lifetimes.

Prior to the characterization of tether stability, we performed a control measurement designed to detect magnetic beads tethered to multiple DNA molecules. This involved applying a pulling force of ∼10 pN and comparing the response of the beads upon clockwise versus counterclockwise magnet rotation of 60 turns. When a single magnetic bead is tethered to the surface via multiple DNA molecules, a significant decrease of the DNA tether extension should be observed in both cases, an effect caused by the wrapping of the molecules about one another (42). Such multiply tethered magnetic beads were excluded from analysis. To evaluate the force-dependent rupture stability of the different types of DNA tethers, we performed dynamic force spectroscopy up to a maximum force of 150 pN, using a constant force loading rate of 10 pN/s. The lifetime analysis for each DNA construct was performed by applying a constant force of 45 pN over the duration of 24 h and detecting the time of tether rupture. For the covalently coupled DNA constructs, this experiment was also repeated at a constant force of 150 pN. All dynamic force spectroscopy and lifetime experiments were carried out in 10 mM TRIS/HCl buffer (pH 7.5, Sigma, USA) containing 100 mM NaCl.

## RESULTS AND DISCUSSION

We systematically characterize our three different chemical anchoring strategies for DNA (Figure 1, ‘Methods’ section) according to criteria designed to test their suitability in single-molecule magnetic tweezers experiments. First, we analyze the non-specific adhesion of functionalized magnetic beads to different surface coatings, as excessive non-specific adhesion may limit the multiplexing efficacy of magnetic tweezers experiments. The effect of different ionic concentrations is taken into account, as magnetic tweezers experiments may require adapted buffer conditions for the study of specific biological processes. Next, we examine the spatial stability of covalently surface-bound polystyrene reference beads to surfaces (Figure 2). Such reference beads are typically employed in single-molecule experiments to correct for instrumental drift. To evaluate the force and lifetime stability of different chemically anchored DNA tethers, we perform force-dependent rupture experiments with pulling forces up to 150 pN and lifetime experiments with durations up to 24 h under constant force loads of 45 (Figure 3) or 150 pN (Table 2). A summary of the quantitative results is supplied in Table 2.

**Table 2.** Maximum applicable pulling forces and median average tether lifetimes at a constant pulling forces of 45 or 150 pN for the three different DNA tethering methods

Construct	Applicable force	Lifetime at 45 pN	Lifetime at 150 pN
Standard	54 ± 11 pN	∼7 min	
Covalent bond at one extremity	150 pN	∼3 h	∼1 min
Covalent bond at both extremities	150 pN	>24 h	>24 h

### Effect of PEG on surface passivation

We characterize the different combinations of functionalized flow cell surfaces and magnetic beads to investigate the presence of non-specific interactions. For quantitative evaluation, we count the number of non-specific adhered magnetic beads per surface area (examples shown in Figure 2A and B) for each surface-bead combination in the presence of 1% (v/v) BSA, which is predominantly used as non-specific adhesion-decreasing adjunct (43). To additionally investigate the effect of ionic strength on surface-bead interactions, we perform these experiments at two different monovalent ion concentrations (50 mM NaCl, 500 mM NaCl) and examine the effect of adding divalent magnesium cations (addition of 7 mM MgCl_2_ to 500 mM NaCl) (Figure 2C). To evaluate statistically our results, we apply an analysis of variances (ANOVA) followed by a Tukey's post-hoc test with a significance threshold level *P < 0.05* (40).

Our experimental results (Figure 2) indicate that when no or solely one PEG crosslinker is used in the different bead-surface combinations, similar degrees of non-specific interactions occur between different functionalized magnetic beads and surface compositions under all tested buffer conditions (Figure 2C). For example, we observe that the standard method of using streptavidin-coated beads and nitrocellulose surfaces results in a relatively low density (∼650 ± 170 beads/mm^2^) of non-specifically adhered beads at low salt conditions (Figure 2A; Figure 2C, gray bar). Under these ionic conditions, PEGylated magnetic beads exposing neutravidin (Figure 2C, red bar) and maleimide (Figure 2C, blue bar) exhibit similar densities (∼710 ± 180 beads/mm^2^ and ∼500 ± 230 beads/mm^2^, respectively) of adhered beads to nitrocellulose surfaces. Comparable densities of adhered magnetic beads are also observed when streptavidin-coated beads are tested on PEG surfaces (Figure 2C, gray hatched bar, (∼720 ± 280 beads/mm^2^). The use of higher salt conditions (500 mM NaCl and 500 mM NaCl supplemented with 7 mM MgCl_2_; Figure 2C) leads to moderately increased bead adhesion for PEGylated beads that expose neutravidin and maleimide (Figure 2C, red and blue bar, respectively), but the adhesion of streptavidin-coated beads to nitrocellulose and PEG surfaces (Figure 2C, gray and gray hatched bars) remain mostly unaffected.

The density of non-specifically adhered beads can be reduced ∼3-fold by switching to the combination of PEG-coated surfaces and PEGylated magnetic beads (Figure 2B). For example, for neutravidin-exposing PEGylated beads tested on PEG surfaces, the bead density is reduced to ∼200 ± 80 beads/mm^2^ (Figure 2C, red hatched bar). Similar results are obtained for maleimide-exposing PEGylated beads tested on PEG surfaces (Figure 2C, blue hatched bars), and overall, the results appear independent of the salt conditions tested. In general, non-specific bead adhesions may result from either electrostatic forces—e.g. Coulomb forces or the van der Waals force—or entropic forces (37,44,45). The use of non-charged PEG crosslinkers provides a spatial separation between the magnetic bead and neighboring surfaces on the order of ∼2 nm per PEG layer (corresponding to the Flory radius of 5 kDa PEG (37)). The Debye length under the ionic conditions tested lies below the provided surface-bead distances of the PEG coatings, suggesting that the introduction of PEG layers does not affect electrostatic interactions except when bead and surface are already in very close proximity. Any reduction in adhesion due to the introduction of PEG layers, which is most pronounced in the case of PEG-coated beads combined with PEGylated surfaces, is therefore likely a result of entropy repulsion, as was also concluded in a previous report that utilized longer, more brush-like PEG layers (37). Remaining adhesion between PEGylated beads and PEG surfaces could possibly result from defects within the PEG-coatings.

### Spatial reference bead fixation stability

We next verify the compatibility of our procedure for the covalent coupling of DNA tethers to surfaces with the stable fixation of so-called reference beads. Position-stable reference beads on sample surfaces are essential to the success of magnetic tweezers measurements (10,46), as they permit the removal of artifacts related to instrumentation and sample drift. A common approach is to affix polystyrene reference beads to nitrocellulose surfaces by non-specific adhesion, as described in the ‘Methods’ section (2,5,7,9,10,21,47). In the case of PEG-coated surfaces, however, this bead attachment method can no longer be applied, as result of adhesion suppression by the PEG polymer layer as described in the previous subsection. An alternative approach is to melt polystyrene beads onto glass surfaces prior to the organochemical surface coating. When surfaces are coated with nitrocellulose, this method guarantees a simple and reliable method for reference bead fixation. In the case of PEG-coated surfaces, however, the required high temperatures damage the ethanolamine and PEG layers, resulting in non-homogenous coverage. In areas of decreased PEG-linker densities this would consequently lead to increased non-specific binding of magnetic beads and biomolecules. We have therefore developed a new anchoring procedure for reference beads that relies on the same accessible carboxylic moieties on surface-anchored PEG crosslinkers employed in the covalent surface attachment of amino-labeled DNA tethers (Figure 1B and C). This method allows the fixation of amine-coated reference beads.

To test the fixation of covalently attached beads, we compare their spatial stability with that of surface-melted polystyrene beads. To do so, we tracked the motions of beads anchored via either approach in three dimensions over 2000 s at 60 Hz (*n* = 25 for both cases). The tracked motions of any individual bead were corrected for instrumental drift in all dimensions by subtracting the coordinates of a second, similarly anchored bead. We analyze the resulting drift-corrected bead movements by computing the corresponding overlapping Allan deviation (48) for averaging times between ∼20 ms and 3 s (Figure 1D). The experimental results for both anchoring strategies display deviations of bead motion in all three dimensions that fall below 13 Å (Figure 1D). Additionally, the differences in the Allan deviations between two approaches for reference bead fixation are <4 Å over the entire averaging time scale (Figure 1D, compare black and red dashed lines). For short averaging times between ∼20 and 100 ms, all bead motions generally remain below 4 Å for all dimensions. Toward higher averaging times up to 3 s, the bead stability decreases globally, with excursions up to 13 Å that result from the increasing influence of instrumental drift. These results demonstrate that both approaches are equally suitable for high-resolution measurements (41,46,49) and, more importantly, that we have provided a protocol for reference bead fixation that is compatible with covalent tethering of DNA tethers.

### Force-dependent extension of linear and DNA hairpin constructs

We now discuss the use of our functionalization process and the addition of PEG-polymers for tethering molecules such as DNA. Before characterizing the overall force- and life-time characteristics of populations of DNA tethers (see 'Force stability and tethter limetimes of linear dsDNA' in 'Results and Discussion' section), we have verified that our newly-introduced DNA anchoring methods do not affect the mechanical properties of individual DNA molecules (23,24,50,51). To do so, we apply force to both individual covalently surface-anchored linear 4.8 kbp dsDNA constructs (Figure 1B) and to 1 kbp DNA hairpin containing constructs. The latter molecules are typically used to study DNA-binding proteins and serve as sensitive and reliable force sensors (49,52,53). We perform the experimental evaluation conducting dynamic force spectroscopy (DFS) measurements (Figure 3A) with a constant force loading of 10 pN/s up to 115 pN.

For both covalently tethered dsDNA (Figure 3B) and hairpin-DNA (Figure 3C), we find that their response to force exhibits the expected behavior. For example, the application of force to a covalently anchored linear dsDNA construct demonstrates regular entropic stretching behavior at pulling forces below 5 pN, followed by enthalpic stretching at higher forces and leading to the observation of the well-described force-induced overstretching transition at ∼60 pN (Figure 3B). The clearly observable overstretching transition is associated with a rapid ∼1.7-fold length increase, in agreement with previous reports (23,51). As expected, the force-extension curve of dsDNA up to the overstretching transition can be well-described by the extensible worm-like chain (WLC) model (50,54). Application of this model to our dsDNA stretching data (Figure 3B, dashed line) yields a bending persistence length of ∼40 nm and a stretching modulus of ∼800 pN, in agreement with previous studies (24,50,55). The application of force to DNA tethers containing a 1-kbp hairpin demonstrates the mechanical unzipping of the double-stranded hairpin structure (Figure 3C). We observe full opening of the 1 kbp hairpin, as witnessed by a length increase of ∼1 μm, at a force of ∼13 pN with, also in agreement with previous studies (11,56,57).

### Force stability and tether lifetimes of linear dsDNA

We now test the coupling stability of the three dsDNA tether anchoring methods by measuring the overall force- and life-time characteristics of populations of DNA tethers. To do so, dsDNA constructs anchored via the different strategies are subjected to increasing forces up to 150 pN. Separately, we conduct lifetime experiments at constant forces of 45 or 150 pN over 24 h. The results of the force-dependent tether stability and the construct lifetimes are summarized in Table 2.

Our experiments indicate that there are significant differences in the force stability and tether lifetimes for dsDNA tethers anchored via the three different approaches (Figure 3), with the common approach of relying on DIG:anti-DIG and streptavidin:biotin interactions (Figure 1A) providing the weakest linkages. The pulling of linear dsDNA tethers coupled using this approach results in average pulling force stabilities of *F*_m__a__x_ ∼55 pN (Figure 3D). We perform lifetime measurements at 45 pN, a force selected because it is ∼20% lower than the average force at which the DIG:anti-DIG coupling fails. Under this force load, this dsDNA construct demonstrates widely distributed lifetimes ranging from a few seconds to ∼40 min. The spread in lifetimes likely reflects the number of contributing DIG:anti-DIG complexes to the dsDNA anchorage (27). On average, however, dsDNA anchored via this common approach last only ∼7 min (Figure 3E).

In stark contrast, the approaches introducing one or more covalent linkages, which in principle provide pulling force stabilities up to nanoNewtons (58), provide vastly-improved force stabilities and lifetimes. For example, dsDNA tethers anchored covalently onto PEGylated surfaces by multiple amide bonds and coupled to magnetic beads via either multiple neutravidin:biotin complexes or a single maleimide:thiol coupling (Figure 1B and C) exhibited no force-dependent tether ruptures even for applied forces as high as 150 pN (Table 2). Similarly, atomic force spectroscopy studies reported pulling force stabilities ≥200 pN for both binding complexes (26,59,60). Concretely, the fact that the maximal force is similarly high in these two cases demonstrates that the neutravidin:biotin coupling does not limit the maximal force that can be applied. Differences between the approaches relying on one versus two covalent linkages (Figure 1B and C) nevertheless appear in the lifetime experiments. These were again performed at a constant force of 45 pN, to compare to the data in Figure 3E. However, it was necessary to increase the duration of the lifetime experiments to 24 h. The dsDNA constructs covalently attached to the PEGylated surfaces and tethered to magnetic beads by several neutravidin:biotin complexes now exhibit a distribution of lifetimes that ranges from ∼1 to 24 h, where the range likely reflects that the presence of differing numbers of neutravidin:biotin binding complexes. On average, their lifetime exhibits a dramatic 30-fold increase to ∼3 h compared to the common attachment strategy (Figure 3F). Impressively, the dsDNA constructs covalently anchored to the PEG-coated surface and covalently tethered to maleimide-modified magnetic beads (Figure 1C) by a single thioether linkage remain stable at this force load over the entire duration of 24 h (Figure 3G). More dramatic differences between these two constructs were observed at a constant force load of 150 pN: here, dsDNA constructs covalently tethered only to PEGylated surfaces demonstrate a lifetime distribution that range from few s to ∼3 h lifetimes, with a median of ∼1 min (Table 2, *n* = 94), while dsDNA constructs covalently tethered at both extremities continue to display lifetimes in excess of 24 h (Table 2, *n* = 28). This result demonstrates that covalent anchorage of both DNA extremities provides the ideal experimental scenario.

The results for the experiments on tether rupture force and lifetime results are summarized in Table 2. The statistical overview clearly demonstrate explicitly the disadvantage of using non-covalent interactions for DNA-anchoring, as the observed lifetimes at high forces are limited to the order of minutes, which drastically impacts the observation time for biophysical experiments. Strategies that employ multiple of such weak linkages can contribute to both force- and life-time stability and can prove useful in certain experiments, but, as our experiments show, eliminating DIG:anti-DIG coupling will enhance the force range while eliminating neutravidin:biotin coupling will enhance the lifetime stability. Indeed, using covalent tethering to both DNA extremities makes it possible to perform magnetic tweezers experiments on DNA molecules that can withstand pulling forces up to 150 pN with lifetimes over 24 h. In such cases, the applicable forces and lifetimes are solely limited by the intrinsic stability of the studied biomolecules or biomolecular complexes.

## CONCLUSIONS

We have demonstrated a significant increase in pulling force stability and lifetimes under force load for DNA tethers by applying covalent chemical tether-anchoring using heterobifunctional PEG crosslinker for magnetic tweezers experiments. By applying this methodology, the limit of lifetime and applicable force will principally be defined by the stability of the biological sample itself. Conveniently, the implementation of biocompatible PEG crosslinkers also provides suppression against non-specific adhesion of magnetic beads and biomolecules on the surface. These advantages provide vastly enhanced high-throughput efficiency in magnetic tweezers experiments and can lead, together with advances in multiplexing capability (8–10) to improved statistics for the description of biomolecular processes. The anchoring method that we present is not limited only to magnetic tweezers: it can be easily applied to other single-molecule techniques, such as optical tweezers and fluorescence-based studies, as well as for e.g. biomaterial engineering and biosensors, where stable biomolecule attachment is required. It can also readily be extended to the binding of proteins, peptides and a wide range of other organic molecules. This development opens new possibilities for single-molecule experiments that probe the stability of biomolecules and interactions under high force, such as DNA-binding protein interactions, protein–protein complexes and protein unfolding studies, amongst others.
